# Supplementary material for: A zebrafish model of chronic heart failure caused by protein aggregation in heart valves
Source: Commun Biol. 2025 Nov 4;8:1520. doi: 10.1038/s42003-025-08882-3 (PMC12586624; doi:10.1038/s42003-025-08882-3)
Supplement: Supplementary file 1 — Supplementary Information [file 42003_2025_8882_MOESM1_ESM.pdf]

Supplementary Figures

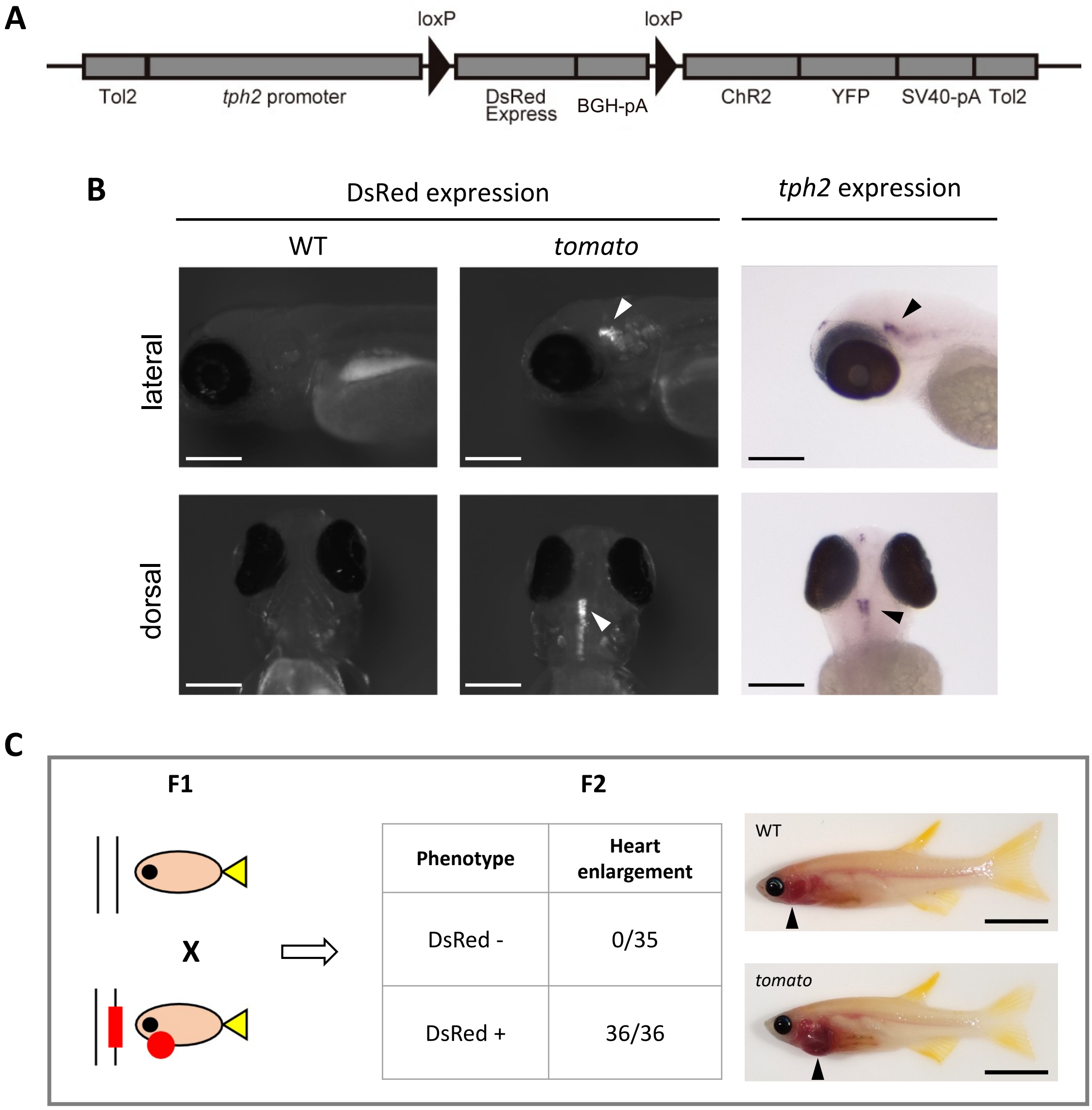

Supplementary Figure 1. Generation of zebrafish *tomato* mutants.

A, The schematics of artificial gene for generating *tomato* fish. DsRed cDNA was placed between a LoxP-flanked cassette with bovine growth hormone polyadenylation (BGH-pA), a specialized termination sequence for protein expression. The *tph2* promotor was employed to drive this transgene. Upon confirmation of successful gene insertion through DsRed expression, *ChR2-YFP* will be expressed under the regulation of the *tph2* promotor following Cre-mediated excision of the DsRed cassette, enabling optogenetic experiments. *SV40-pA*, SV40 polyadenylation sequence. **B,** The DsRed expression pattern resembles the *tph2* expression pattern in the brain at 3 dpf. Schematic representation of DsRed expression under a fluorescence microscope from lateral and dorsal views (left). Diagram of *tph2* expression pattern in the brain detected by whole-mount in situ hybridization (right). White arrows point to DsRed expression, black arrows point to *tph2* expression. Scale bar, 200μm. **C,** Schematic diagram of breeding experiment. Black arrows point to zebrafish hearts. Scale bar, 5mm.

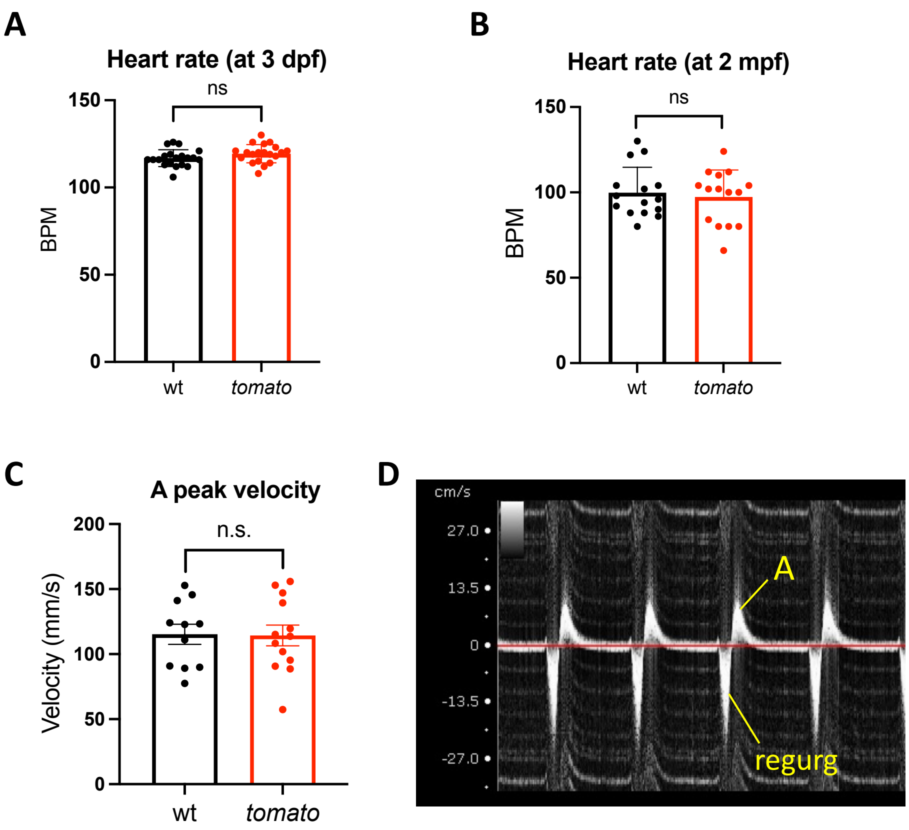

Supplementary Figure 2. A, Heart rates measured in WT and *tomato* fish at 3 days post-fertilization (dpf). N=20 fish in each group. BPM, beats per minute. **B,** Heart rates measured in WT and *tomato* fish at 2 months post-fertilization (mpf). N=15 fish in each group. **C**, The velocity in late diastole was measured from WT (N=11) and *tomato* (N=13) fish at 3 mpf. **D**, PWD inflow in *tomato* fish shows E wave absence. The arrows indicate the late diastole wave (A), and regurgitation (regurg). Y axis represents velocity in cm/s. Data are presented as mean ± SEM. Statistics: unpaired 2-tailed student t test in (**A**, **B** and **C**).

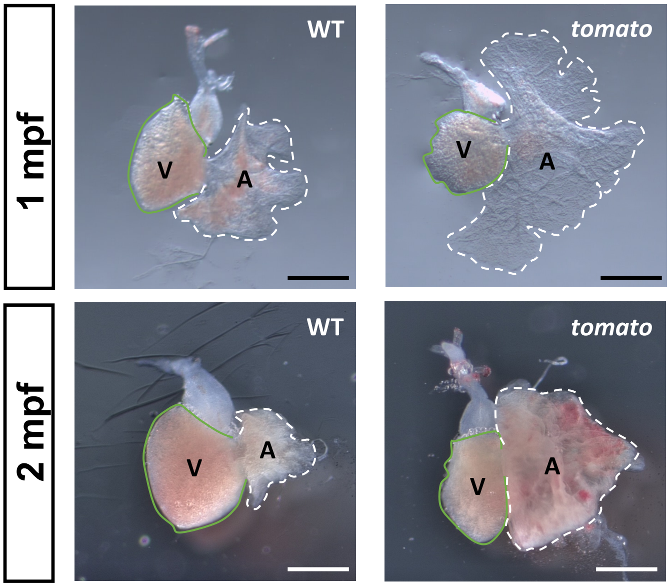

Supplementary Figure 3. Representative images of hearts extracted from wild-type (WT) and *tomato* zebrafish at 1 month post-fertilization (mpf) and 2 mpf. White dotted lines: atrium. Green lines: ventricle. Scale bars, 500μm. A: atrium, V: ventricle.

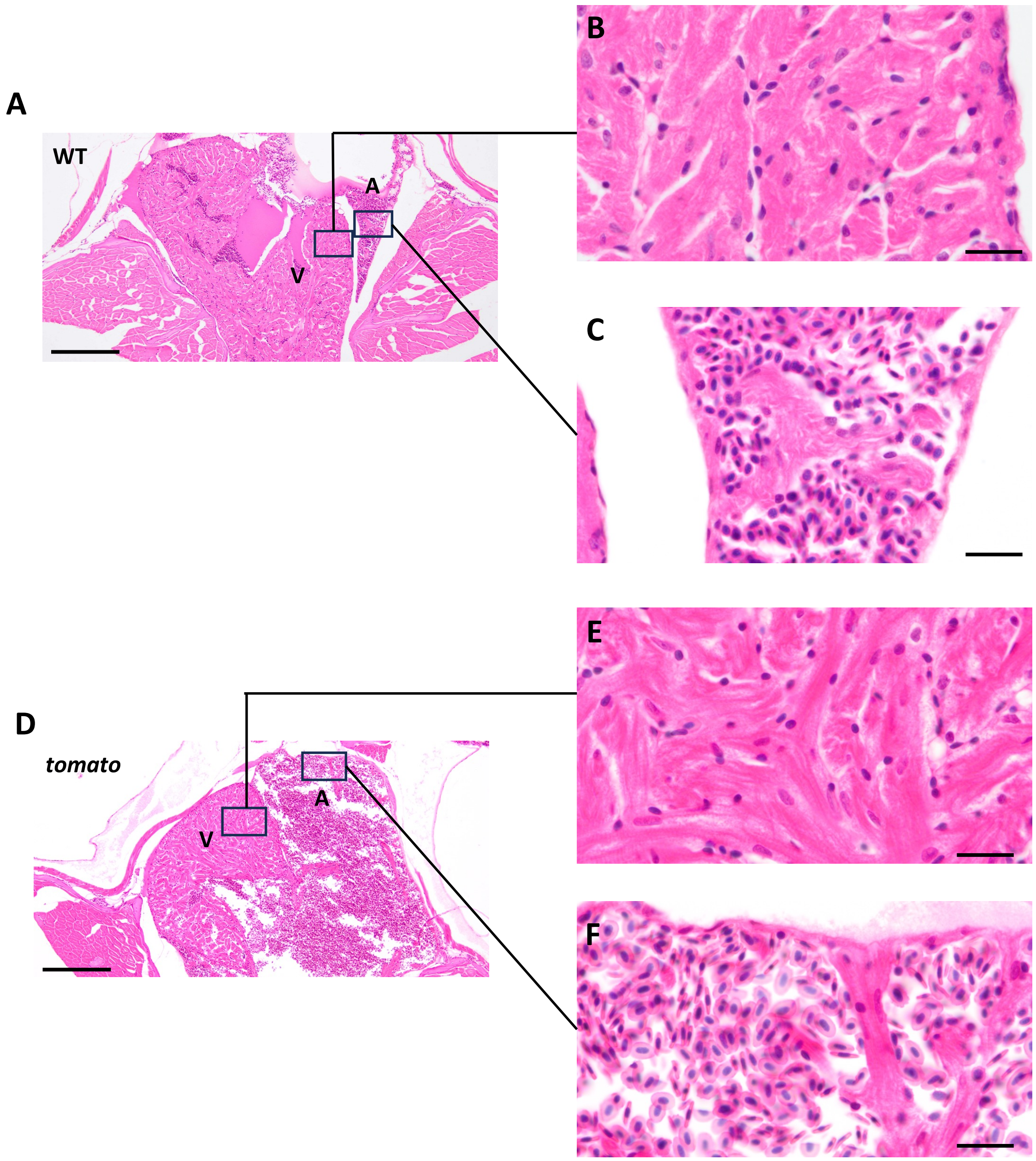

Supplementary Figure 4. Magnified H&E stained images of atrial and ventricular myocardium in adult zebrafish. Representative coronal heart sections from WT (A-C) and *tomato* (D-F). **A,** Overview of the WT heart. Boxed areas corresponding to higher magnification images of ventricle (**B**) and atrium (**C**). D, Overview of the *tomato* heart. Boxed areas corresponding to higher magnification image of ventricle (**E**) and atrium (**F**). Scale bar, 200µm in **A** and **D**; 20µm in **B**, **C**, **E** and **F**. A: atrium, V: ventricle.

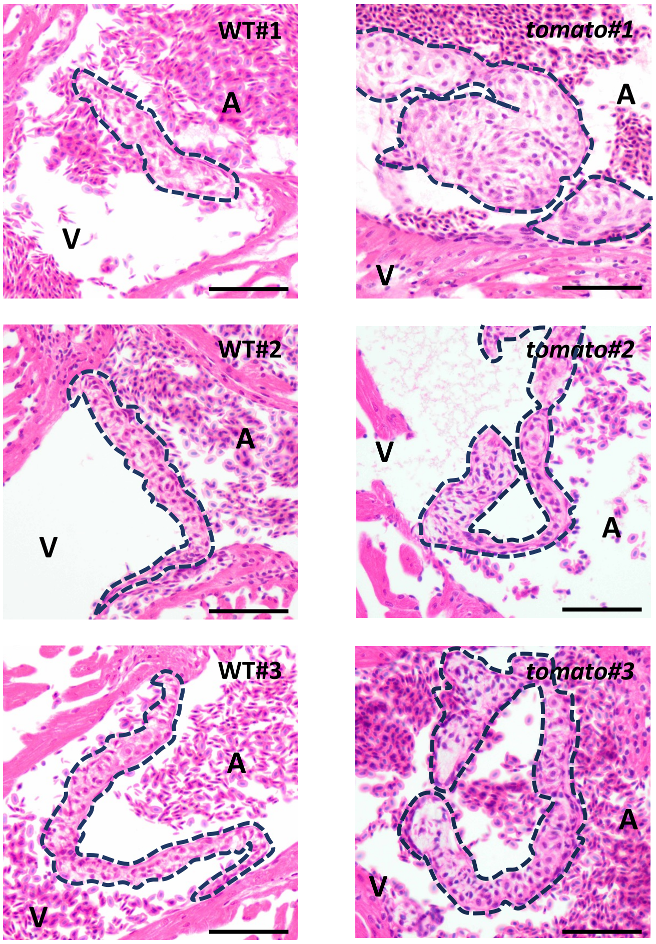

Supplementary Figure 5. Magnified images of AV valve from additional 3 samples in WT and *tomato* zebrafish, corresponding to sample shown in Figure 3H-I. Dark blue dotted lines: atrioventricular (AV) valves. Scale bar, 50μm. A: atrium, V: ventricle.

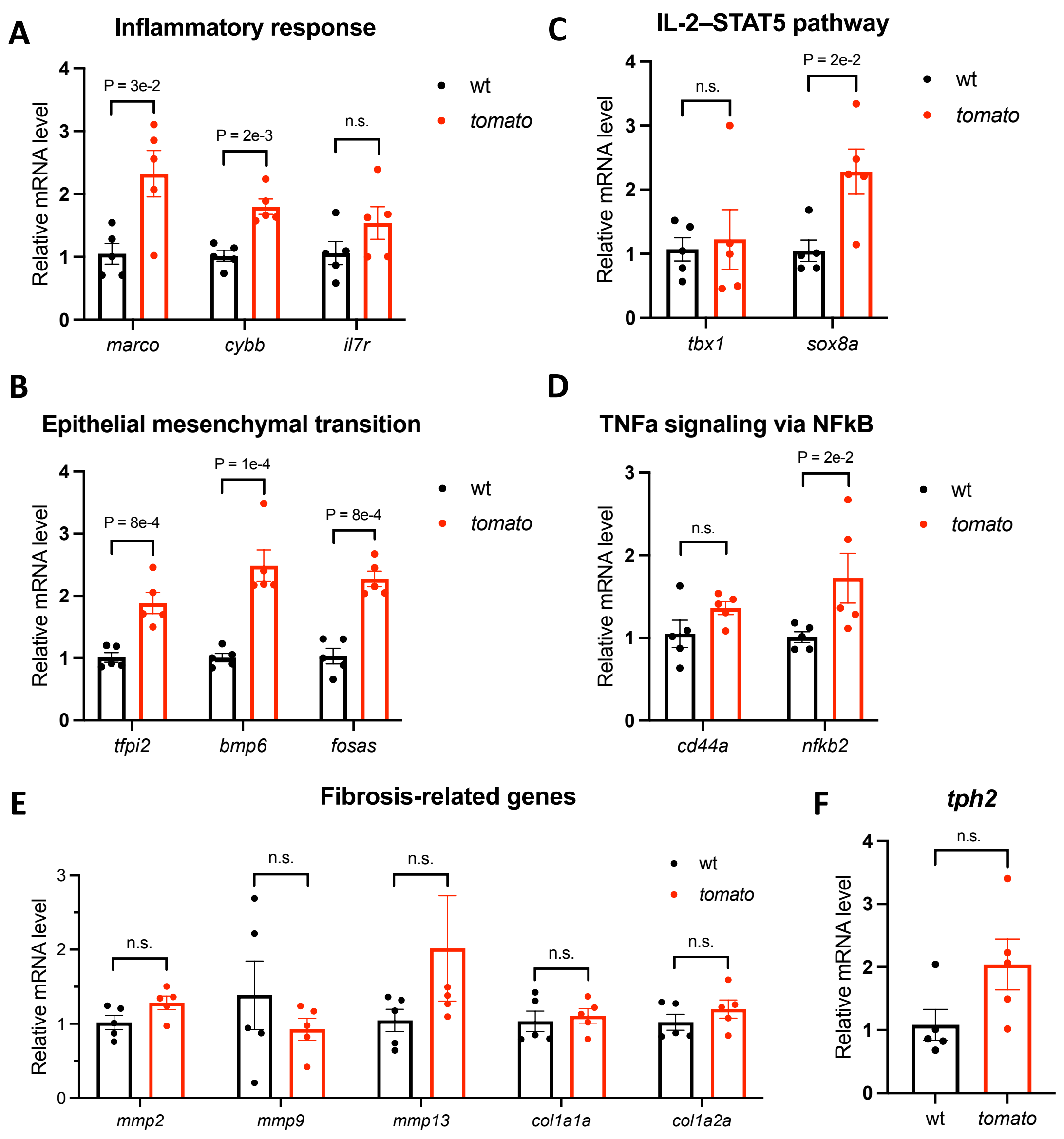

Supplementary Figure 6. A, Relative expression of *marco, cybb, il7r* in inflammatory response. **B,** Relative expression of *tfpi2, bmp6, fossa* in Epithelial mesenchymal transition. **C,** Relative expression of *tbx1* and *sox8a* in IL-2–STAT5 pathway. **D,** Relative expression of *cd44a, nfkb2* in TNFa signaling via NFkB pathway. **E,** Relative expression of *mmp2*, *mmp9*, *mmp13*, *col1a1a*, and *col1a2a.* **F,** Relative expression of *tph2*. The expression level of mRNA from WT and *tomato* fish hearts at 3mpf were normalized to *actb1*. N = 5 fish in each group. Data are presented as mean ± SEM. Statistics: Multiple unpaired t test with the Holm-Sidak correction in (**A**–**E**) and unpaired 2-tailed student t test in (**F**).

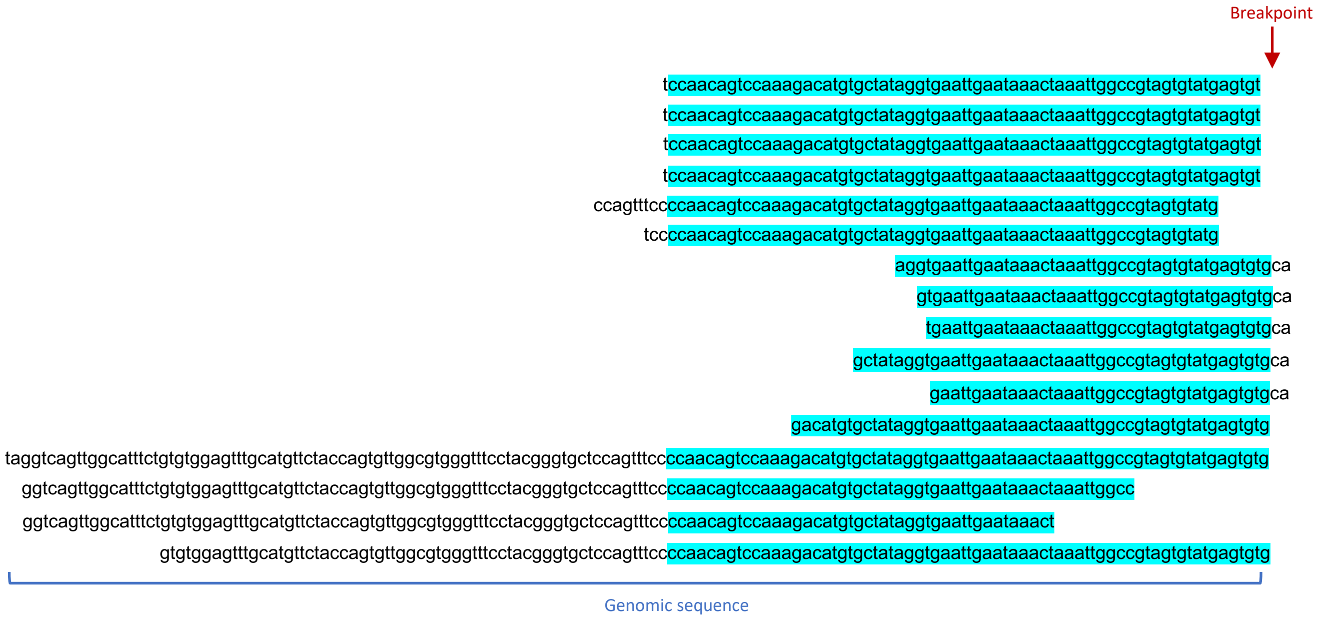

Supplementary Figure 7. Alignment of all whole genome sequencing (WGS) reads. The shared genomic sequence segment among reads is highlighted in blue. Red arrows indicate the breakpoint.

Supplementary Table 1. Lists of hallmark gene set with significantly change in tomato zebrafish heart.

ID	Hallmark gene set	Group	P-value	P-adjust
M5890	TNFa signaling via NFkB	Up	4.970.E-07	2.390.E-05
M5930	Epithelial mesenchymal transition	Up	5.020.E-05	1.204.E-03
M5932	Inflammatory response	Up	1.472.E-04	2.356.E-03
M5947	IL2-STAT5 signaling	Up	2.002.E-03	2.402.E-02
M5925	E2F targets	Up	4.226.E-03	3.484.E-02
M5953	KRas signaling up	Up	4.355.E-03	3.484.E-02
M5909	Myogenesis	Down	4.274.E-04	1.452.E-02
M5935	Fatty acid metabolism	Down	6.752.E-04	1.452.E-02
