# Supplementary material for: A zebrafish model of chronic heart failure caused by protein aggregation in heart valves
Source: Commun Biol. 2025 Nov 4;8:1520. doi: 10.1038/s42003-025-08882-3 (PMC12586624; doi:10.1038/s42003-025-08882-3)
Supplement: Supplementary file 2 — Description of Additional Supplementary Materials [file 42003_2025_8882_MOESM2_ESM.pdf]

Description of Additional Supplementary Files

File name: Supplementary Data 1

Description: The source data for Figures 2, 3 and supplementary figure 2 (S2), supplementary figure 6 (S6)

File name: Supplementary Data 2

Description: Lists of expressed genes significantly changed in tomato zebrafish heart.

File name: Supplementary File 1

Description: The complete sequence of the plasmid. (Viewable with ApE software, https://jorgensen.biology.utah.edu/wayned/ape/)

File name: Supplementary File 2

Description: The sequence of identified PCR amplified product, including ~200bp genomic sequence and 300 bp transgene sequence. (Viewable with ApE software)

File name: Supplementary Movie 1

Description: wt heart image under brightfield at 14dpf.

File name: Supplementary Movie 2

Description: wt heart image under RFP fluorescence at 14dpf.

File name: Supplementary Movie 3

Description: tomato heart image under brightfield at 14dpf.

File name: Supplementary Movie 4

Description: tomato heart image under RFP fluorescence at 14dpf.
